# Analysis of Regulatory Mechanism of AcrB and CpxR on Colistin Susceptibility Based on Transcriptome and Metabolome of Salmonella Typhimurium

**DOI:** 10.1128/spectrum.00530-23

**Published:** 2023-06-26

**Authors:** Ya-Jun Zhai, Pei-Yi Liu, Xing-Wei Luo, Jun Liang, Ya-Wei Sun, Xiao-Die Cui, Dan-Dan He, Yu-Shan Pan, Hua Wu, Gong-Zheng Hu

**Affiliations:** a College of Veterinary Medicine, Henan Agricultural University, Zhengzhou, China; b Zhengzhou Animal Husbandry Bureau, Zhengzhou, China; c Henan Institute of Science and Technology, Xinxiang, China; Universidad Andres Bello

**Keywords:** colistin susceptibility, AcrB, CpxR, transcriptome, metabolome

## Abstract

With the increasing and inappropriate use of colistin, the emerging colistin-resistant isolates have been frequently reported during the last few decades. Therefore, new potential targets and adjuvants to reverse colistin resistance are urgently needed. Our previous study has confirmed a marked increase of colistin susceptibility (16-fold compared to the wild-type Salmonella strain) of *cpxR* overexpression strain JSΔ*acrB*Δ*cpxR*::*kan*/p*cpxR* (simplified as JSΔΔ/p*R*). To searching for potential new drug targets, the transcriptome and metabolome analysis were carried out in this study. We found that the more susceptible strain JSΔΔ/p*R* displayed striking perturbations at both the transcriptomics and metabolomics levels. The virulence-related genes and colistin resistance-related genes (CRRGs) were significantly downregulated in JSΔΔ/p*R*. There were significant accumulation of citrate, α-ketoglutaric acid, and agmatine sulfate in JSΔΔ/p*R*, and exogenous supplement of them could synergistically enhance the bactericidal effect of colistin, indicating that these metabolites may serve as potential adjuvants for colistin therapy. Additionally, we also demonstrated that AcrB and CpxR could target the ATP and reactive oxygen species (ROS) generation, but not proton motive force (PMF) production pathway to potentiate antibacterial activity of colistin. Collectively, these findings have revealed several previously unknown mechanisms contributing to increased colistin susceptibility and identified potential targets and adjuvants for potentiating colistin treatment of Salmonella infections.

**IMPORTANCE** Emergence of multidrug-resistant (MDR) Gram-negative (G^-^) bacteria have led to the reconsideration of colistin as the last-resort therapeutic option for health care-associated infections. Finding new drug targets and strategies against the spread of MDR G^-^ bacteria are global challenges for the life sciences community and public health. In this paper, we demonstrated the more susceptibility strain JSΔΔ/p*R* displayed striking perturbations at both the transcriptomics and metabolomics levels and revealed several previously unknown regulatory mechanisms of AcrB and CpxR on the colistin susceptibility. Importantly, we found that exogenous supplement of citrate, α-ketoglutaric acid, and agmatine sulfate could synergistically enhance the bactericidal effect of colistin, indicating that these metabolites may serve as potential adjuvants for colistin therapy. These results provide a theoretical basis for finding potential new drug targets and adjuvants.

## INTRODUCTION

As a major cause of foodborne gastroenteritis, the Gram-negative (G^-^) bacterial pathogen Salmonella enterica serovar Typhimurium (Salmonella Typhimurium) is generally transmitted to humans through oral ingestion of contaminated food or water (1, 2). With the constantly emerging of new drugs to manage infections, new problems that increasing resistance rates to β-lactams, quinolones, and aminoglycosides among MDR G^-^ bacteria come after, which has led to the revival of colistin in recent years (3). Colistin is potent bactericidal antibiotic against MDR G^-^ bacterial infections (4). Unfortunately, the increased and disproportionate use of colistin has been followed by an increase in colistin-resistant bacteria (5, 6).

The two-component system (TCS) CpxAR harbors two proteins, a sensor histidine kinase CpxA and a cognate response regulator CpxR. The phosphorylated CpxR (CpxR-P) could function as a transcription factor to regulate transcription of target genes (7). The drug efflux pump AcrAB-TolC belongs to the resistance nodulation division (RND) superfamily, and consists of three parts: an inner-membrane protein AcrB, a periplasmic adapter protein AcrA, and an outer membrane protein TolC. AcrB is responsible for the extrusion of toxic compounds, including clinically important antibiotics (8). Several reports focusing on TCS CpxAR, AcrAB-TolC efflux, and antibiotic resistance have indicated that CpxR and AcrAB were responsible for confering resistance to a wide variety of antibiotics by expelling antibiotics and other compounds from the bacterial cell (9–11). Our previous study has indicated that the MIC of colistin for JSΔΔ/p*R* was markedly reduced by 16-fold compared to the wild-type Salmonella strain JS, and also demonstrated that TCS CpxAR could increase the antibacterial activity of colistin against Salmonella through the MgrB-PhoPQ-PmrD-PmrAB-PmrC/H pathway (12).

Omics technologies have provides an opportunity for the identification of specific antimicrobial agents (13). For instance, a study manifested that the Escherichia coli and Salmonella Typhimurium strains with AcrB mutantion showed mostly different metabolic responses relative to those in the wild type, and suggesting that oxidized fatty acids were potential native substrates of AcrB (14). Besides, transcriptomic and metabolomic data could decipher the complex regulation on gene and metabolic responses to various types of environmental niches, as accumulated observations implying that the alterations in metabolic pathways of amino acid, nucleotide, lipopolysaccharide, or tricarboxylic acid (TCA) cycle were implicated in the survival of bacteria cells in acid/oxidative stress, ionizing radiation exposure or mutation pressures (15–18).

Although the underlying molecular basis of the increased colistin susceptibility has been preliminarily explored in our previous research, the comprehensive analysis of transcriptomics and metabolomics in JSΔΔ/p*R* remain unknown. In this context, combined transcriptome and metabolome analysis was preformed to further clarify the gene–metabolite networks in the more susceptible Salmonella Typhimurium strain JSΔΔ/p*R*. Importantly, we demonstrated that exogenous supplement of citrate, α-ketoglutaric acid, and agmatine sulfate could synergistically enhance the bactericidal effect of colistin. Furthermore, we also demonstrated that AcrB and CpxR could target the ATP and reactive oxygen species (ROS) generation, but not proton motive force (PMF) production to potentiate antibiotic activity of colistin. These results revealed several previously unknown regulatory mechanisms of AcrB and CpxR on the colistin susceptibility and lay the foundations for further drug development.

## RESULTS AND DISCUSSION

### AcrB and CpxR modulate the genetic profile of Salmonella Typhimurium.

**FIG 1:**
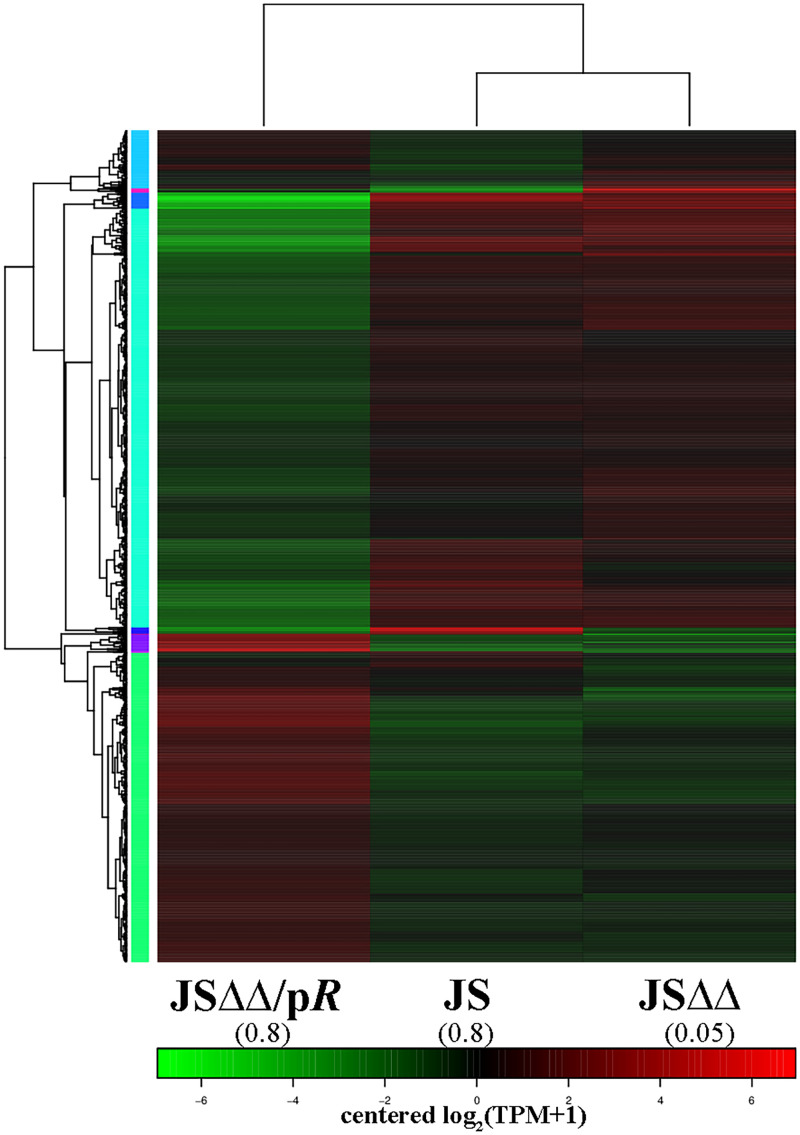
Cluster analysis of differentially expressed genes by TPM value. Each row represents a gene and each column represents a sample. The dendrogram across the top is a tree diagram of sample clustering. The dendrogram on the left illustrates clustering of phenotypes. Red color indicates the upregulated genes, while green color indicates the downregulated genes. The color shades vary with the value of log_2_(TPM + 1). Values in brackets represent the MICs of colistin for JS (0.8 mg/L), JSΔΔ (0.8 mg/L), and JSΔΔ/p*R* (0.05 mg/L).

**FIG 2:**
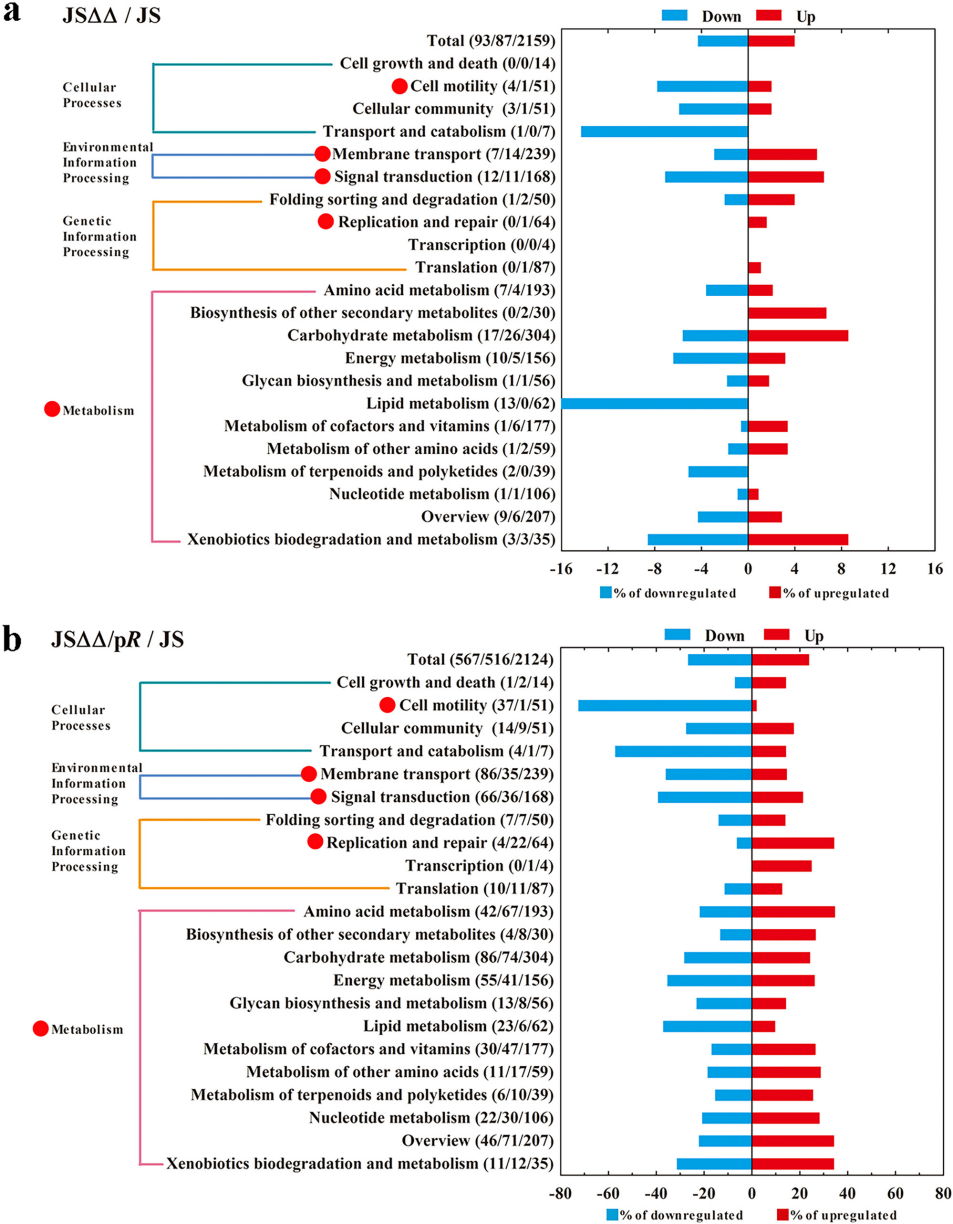
KEGG pathway analysis of differentially transcribed genes in the JSΔΔ versus JS group (a) and JSΔΔ/p*R* versus JS group (b). The name and number of downregulated (blue), upregulated (red) genes in each pathway were indicated in parentheses on the left (down/up/total). Highlighted with red circles are the pathways that SDEGs mainly enriched. The MICs of colistin for JS, JSΔΔ, and JSΔΔ/p*R* were 0.8, 0.8 and 0.05 mg/L, respectively.

In this paper, a comparative analysis was performed within each pair of the three groups JS, JSΔΔ, and JSΔΔ/p*R*. In total, we found 559 (194 up, 365 down), 2,547 (720 up, 1,827 down), and 2,736 (865 up, 1,871 down) differentially expressed genes in JSΔΔ versus JS, JSΔΔ/p*R* versus JSΔΔ, and JSΔΔ/p*R* versus JS, respectively. The cluster analysis showed that the JSΔΔ/p*R* group had a transcription profile different from those of the JS and JSΔΔ groups (Fig. 1). The differentially regulated genes could be classified into four major functional KEGG pathways: (i) cellular processes; (ii) environmental information processing; (iii) genetic information processing; and (iv) metabolism (Fig. 2). KEGG analyses revealed that AcrB and CpxR could modulate the genetic profile of Salmonella Typhimurium, because compared to the parent strain JS, the gene expression of JSΔΔ and JSΔΔ/p*R* were perturbed in various degrees. In general, the significantly differentially expressed genes (SDEGs) with KEGG annotation were more abundant in JSΔΔ/p*R* versus JS (1,094 genes) than that of JSΔΔ versus JS (180 genes), and they were mainly enriched in cell motility (downregulated), membrane transport (up- and downregulated), signal transduction (up- and downregulated), replication and repair (upregulated), and pathways in metabolism (up- and downregulated) in JSΔΔ/p*R*.

### The virulence-related genes were mostly downregulated in JSΔΔ/pR.

Herein, we listed the top 20 significantly up- or downregulated genes of the three groups according to the log_2_FC in the Table S1. Generally, compared to JS, the top 20 SDEGs in JSΔΔ were metabolism-related genes, for example the propanediol utilization and ethanolamine utilization-related genes. However, compared to JS or JSΔΔ, transcriptional changes in JSΔΔ/p*R* occurred not only in metabolism-related genes, but also in virulence-related genes (mostly downregulated), such as *invF*, *invE*, *spaM*, *fimW*, etc. The virulence-related genes included pathogenicity island genes (*invG*, *invE*, *invF*, *sipA*, *sipB*, *sipC*, *sipD*, *prgJ*, *prgI*), cell invasion protein gene (*spaM*), and fimbrial genes (*fimW*, *fimC*, *fimA*). The pathogenicity island genes were required for Salmonella invasion of host cell and aid in intracellular survival and replication (19). Mutations in *invA*, *invC*, and *invG* rendered Salmonella defective in their ability to invade cultured epithelial cells (20, 21). Gene *spaM* encode protein involved in cell invasion (22). Type I fimbriae is known to play a role in Salmonella pathogenicity by facilitating adhering to and invasion of intestinal epithelial cells (23). FimW has been reported as a negative regulator of type I fimbriae genes (24). Genes *fimC* and *fimA* are required for the biogenesis of type I fimbriae, and FimA is found to be the major subunit of the type I fimbriae (25). Thus, decreased *fimW* expression may result in enhaced *fimC*, *fimA* transcription, and *fimW* mutant Salmonella strain were demonstrated to upregulated the *fimA* expression (24). However, in this paper, we detected simultaneous reduction of *fimW*, *fimC*, and *fimA*, which may result from the cross-regulation role of FimY and FimZ (26). Hence, these data indicated that the more susceptible strain JSΔΔ/p*R* may exhibit impaired adhesion and invasiveness.

### Transcriptional changes occured in both colistin resistance-related genes and numerous other TCSs pathways.

**FIG 3:**
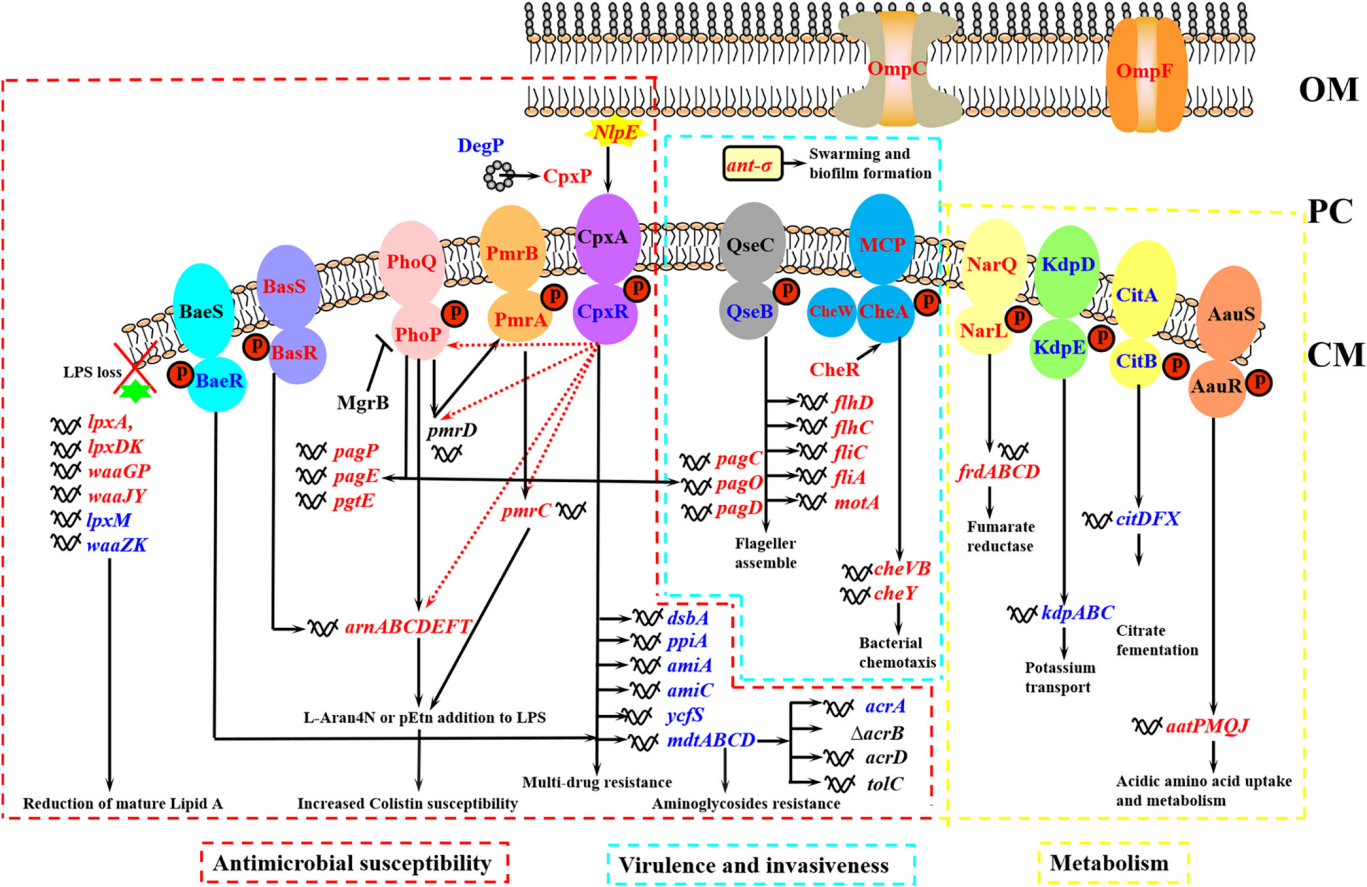
Networks of the SDEGs included in colistin susceptibility, virulence, and metabolism pathways in JSΔΔ/p*R* versus JSΔΔ and JSΔΔ/p*R* versus JS groups. OM, PS, and CM represents outer membrane, periplasmic space, and cytoplasmic membrane, respectively. The red font, blue font, or black font of gene names indicated significantly downregulation, upregulation, or no significant change, respectively. Red dotted arrows between *cpxR* and colistin resistance-related genes (*phoPQ*, *pmrC*, *pmrD*, *arnB*) indicated the results in our previous paper that CpxR could markedly downregulate the transcriptional levels of these genes (12). The MICs of colistin for JS, JSΔΔ and JSΔΔ/p*R* were 0.8, 0.8, and 0.05 mg/L, respectively.

We summarized and drawn the networks of the SDEGs included in TCSs pathways, cationic antimicrobial peptide (CAMP) resistance pathway and lipopolysaccharide biosynthesis pathway in JSΔΔ/p*R* versus JSΔΔ and JSΔΔ/p*R* versus JS groups. As shown in Fig. 3, the SDEGs were annotated and divided into three categories: colistin susceptibility, virulence, and metabolism. In terms of colistin susceptibility, the transcription levels of colistin resistance-related genes (CRRGs), such as TCSs genes *basSR*, *phoPQ*, *pmrAB* and the downstream genes *arnABCDEFT* operon (*arnB* also known as *pmrH*), *pagP*, *pmrC*, *mdtABCD*, were all significantly downregulated, which was reported to play important roles in the process of lipid modification especially the modification of pEtN and L-Ara4N (6). These results were in conformity with the electrospray ionization mass spectrometry (ESI-MS) analysis, which also demonstrated a reduction of pEtN and L-Ara4N modification of lipid A in the colistin susceptible Salmonella Typhimurium strain JSΔΔ/p*R* (Fig. S1). Therefore, the detection of a markedly MIC reduction (16-fold) of colistin for JSΔΔ/p*R* in our previous study were closely related to the reduction of L-Ara4N and pEtN modification of lipid A species (12). Besides, we also figured out the transcriptional changes of TCSs pathways. QseCB cascade have functions associated virulence as well as in flagella biosynthesis (27). The TCS MCP-CheA-CheW consists of methyl-accepting chemotaxis proteins (MCPs), histidine kinase (CheA), and scaffolding protein (CheW). Phosphorylated CheY diffuses to the flagellar motor and results in a change in the direction of cell movement (28). The metabolism-related TCSs NarQL, KdpDE, CitAB, and AauSR were associated with the fumarate reductase, potassium transport, citrate fermentation, acidic amino acid uptake, and metabolism (29–32). These transcriptional changes of above metabolism-related genes and virulence-related genes may be a compensatory reaction. The results remind us to take a broader, holistic view of our research on antibiotic resistance, which should not only take into account the changes of resistance or susceptibility itself, but also the changes in metabolism and virulence of bacteria. There may be a new approach to the treatment of MDR bacteria in these compensatory changes.

### More significantly differential metabolites were detected in JSΔΔ/pR.

In view of the obvious perturbation of metabolic pathways-related genes in JSΔΔ/p*R*, and meanwhile, in consideration of reports that deletion or inhibition of *acrB* may cause the accumulation of antibiotics and harmful metabolites (14), we are curious that whether *acrB* deletion and *cpxR* overexpression in JSΔΔ/p*R* could also increase the accumulation of harmful substances and ultimately led to the increased colistin susceptibility of JSΔΔ/p*R*. We first detected whether the intracellular accumulation concentration of colistin was increased by liquid chromatography tandem mass spectrometry (LC-MS/MS). As shown in the Fig. S2, compared to JS or JSΔΔ, colistin as well as the reported high/low-accumulating drugs, ciprofloxacin and rifampicin, were all significantly accumulated in JSΔΔ/p*R.* Consisting with the mechanism of “direct antibacterial colistin activity” reviewed by El-Sayed Ahmed et al. (33), we considered that the increased uptake of colistin in JSΔΔ/p*R* was responsible for the increased bactericidal effect of colistin.

**FIG 4:**
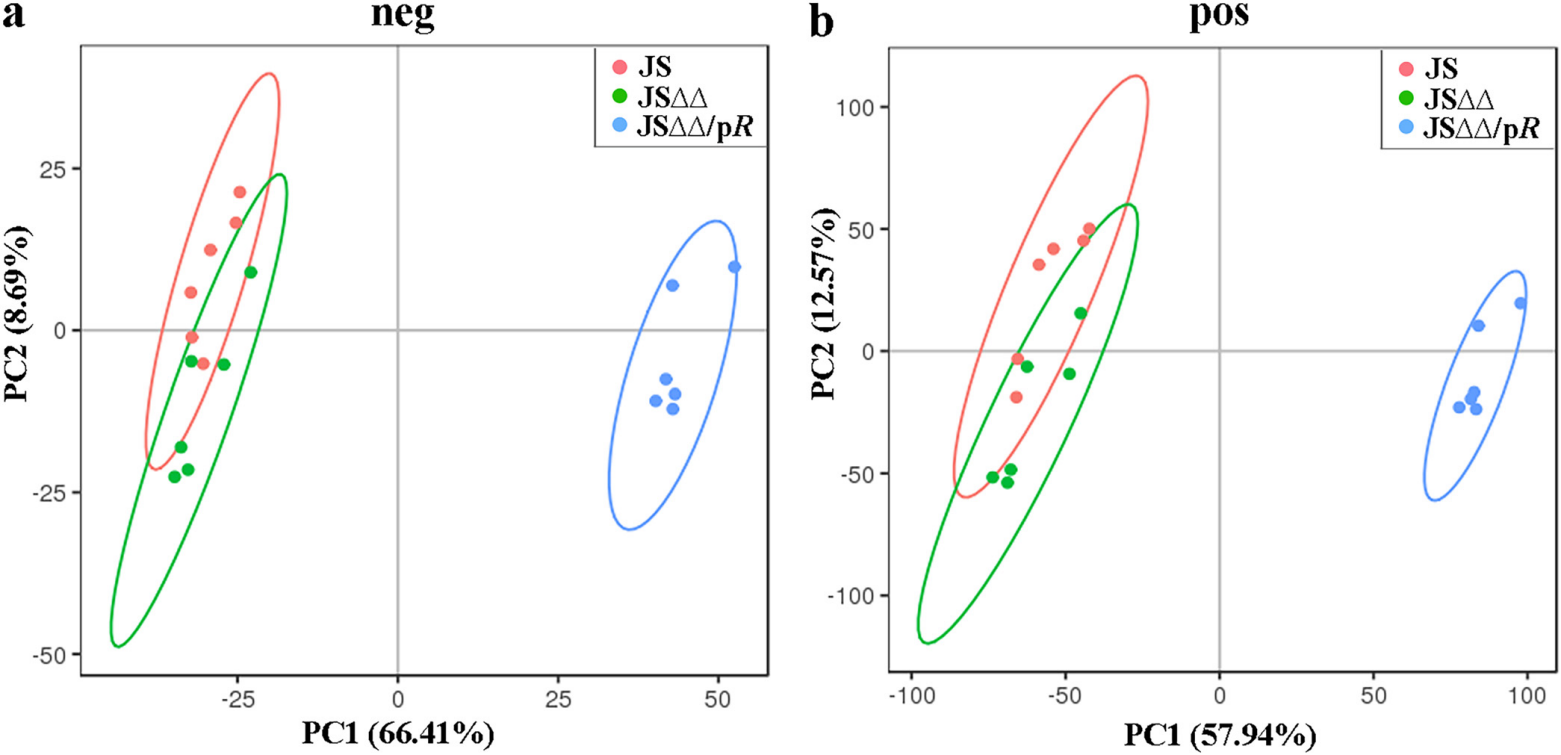
Principal-component analysis of JS, JSΔΔ, and JSΔΔ/p*R*. Metabolites were analyzed by LC-MS/MS in negative mode (a) and positive mode (b). Each dot represents a biological replicate of each strain. The MICs of colistin for JS, JSΔΔ, and JSΔΔ/p*R* were 0.8, 0.8, and 0.05 mg/L, respectively.

The metabolome analysis were subsequently performed between JSΔΔ versus JS, JSΔΔ/p*R* versus JSΔΔ, and JSΔΔ/p*R* versus JS groups. Overall, only 40% of the detected metabolites in these strains were identifiable, and there were more significantly differential metabolites (SDMs) in JSΔΔ/p*R* than JS and JSΔΔ (Table S2). The principal-component analysis (PCA) were carried out and these strains formed two separate clusters, in which JSΔΔ clustered tightly with the wild-type strain JS (Fig. 4). These results indicated that JSΔΔ/p*R* was metabolically different form JS and JSΔΔ.

We then performed a KEGG pathway analysis among these SDMs. A total of 45 metabolic pathways were significantly perturbed (*P < *0.05) in JSΔΔ/p*R* compared with JS or JSΔΔ, embodying in carbon metabolism, pentose phosphate pathway, amino acid metabolism (cysteine, methionine, arginine, histidine metabolism, etc.), TCA cycle, nucleotide (purine and proline) metabolism, and some other metabolism (Table S3). But it should be noted that many SDMs were involved in multiple metabolic networks. For example, pyruvic acid was simultaneously included in pentose phosphate pathway, TCA cycle, biosynthesis of amino acids, and so on. Therefore, by comprehensive analysis of metabolomics, transcriptomics, and previous reports, we concentrated specifically on eight pathways that changed both at transcriptional and metabolic levels (Table 1).

**TABLE 1:** The eight pathways that changed both at transcriptional and metabolic levels^*a*^

Pathway name	Pathway ID	JSΔΔ/p*R* vs. JSΔΔ		JSΔΔ/p*R* vs. JS		JSΔΔ vs. JS		Total
		Neg	Pos	Neg	Pos	Neg	Pos	
Tricarboxylic acid (TCA) cycle	map00020	3	3	2	3	0	0	20
Oxidative phosphorylation	map00190	0	2	0	0	0	0	16
Arginine and proline metabolism	map00330	0	7	0	4	0	0	78
Cysteine and methionine metabolism	map00270	2	6	2	7	0	0	61
Biosynthesis of amino acids	map01230	4	11	4	10	0	0	128
Histidine metabolism	map00340	0	5	2	5	1	0	47
Purine metabolism	map00230	0	7	3	7	0	2	95
Pyrimidine metabolism	map00240	2	5	3	3	0	0	65

a The MICs of colistin for JS, JSΔΔ, and JSΔΔ/p*R* were 0.8, 0.8, and 0.05 mg/L, respectively.

### Exogenous citrate and α-ketoglutaric acid potentiated the colistin-mediated killing of Salmonella.

**FIG 5:**
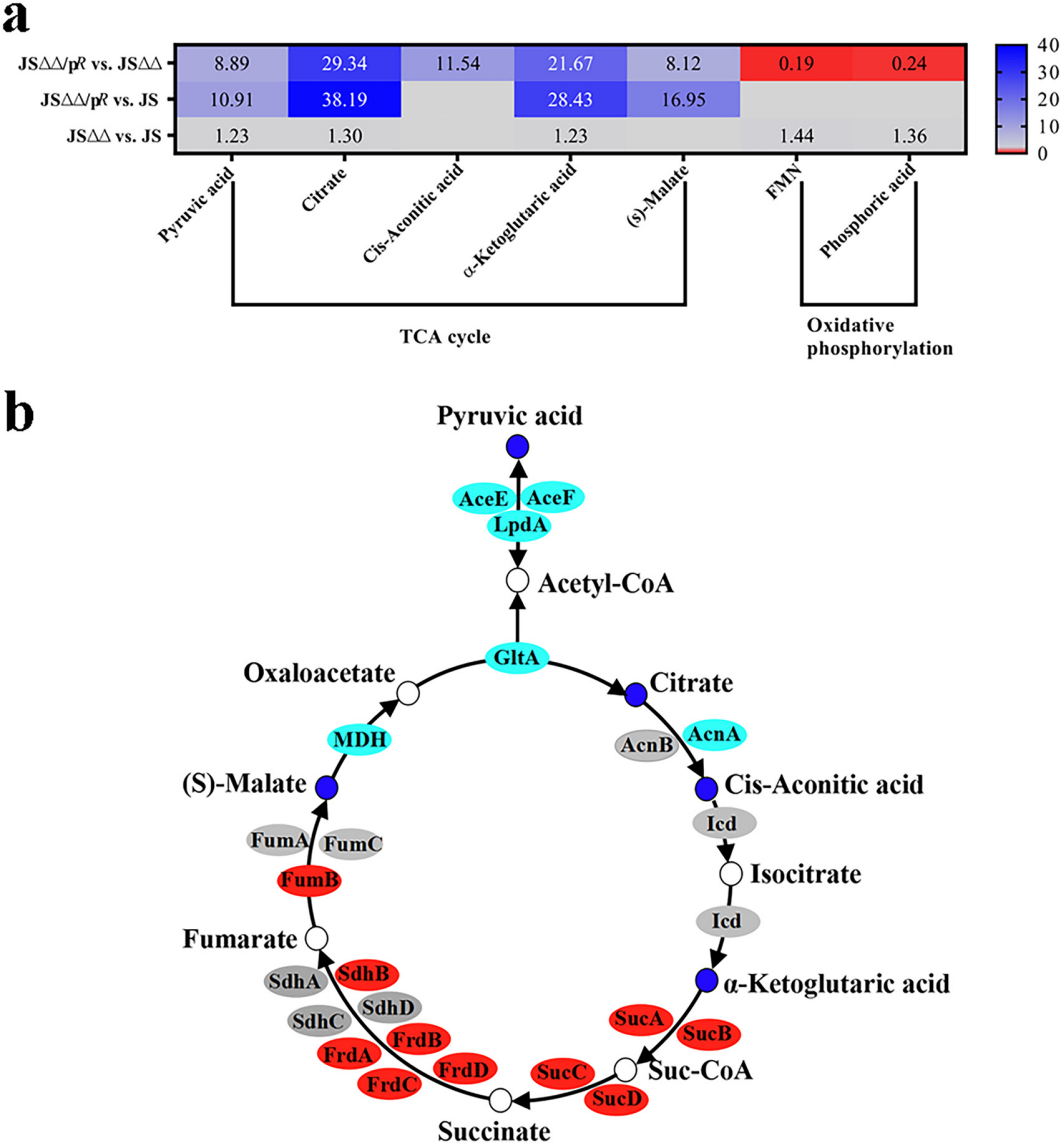
The SDMs detected in TCA cycle and oxidative phosphorylation of JSΔΔ versus JS, JSΔΔ/p*R* versus JSΔΔ, and JSΔΔ/p*R* versus JS groups. (a). Combined transcriptome and metabolome analysis for TCA cycle of JSΔΔ/p*R* versus JS and JSΔΔ/p*R* versus JSΔΔ groups (b). Labels in each square indicate the fold changes of corresponding metabolites and squares without label indicate the data are not credible (*P > *0.05, VIP < 1). Small circles represent metabolites, ovals represent genes. Background colors indicate the relative concentration of the respective metabolite or gene, red = decreased, dark/light blue = increased, yellow = strikingly increased. Gray background indicates indifferent concentrations with *P* < 0.05 and VIP ≥ 1. The MICs of colistin for JS, JSΔΔ, and JSΔΔ/p*R* were 0.8, 0.8, and 0.05 mg/L, respectively.

The TCA cycle, as the common and hub metabolic pathway of carbohydrate lipid and amino acid metabolism, is an integral part for efficient bacterial metabolism in changing environment (18). Recently, numerous studies revealed that metabolic slowdown were associated with the drug-tolerant state of bacteria, and boosting the TCA cycle could alter the metabolic state, which thereby restoring antibiotic sensitivity (34, 35). Surprisingly, we found that the more susceptible strain JSΔΔ/p*R* exhibited a significant increase in several key metabolites of TCA cycle, compared to that of JS or JSΔΔ (Fig. 5). The pyruvic acid, citrate, cis-aconitic acid, α-ketoglutarate, and (s)-malate level exhibited a substantial increase, being elevated 8 to 38-fold in JSΔΔ/p*R* strain (Fig. 5).

**FIG 6:**
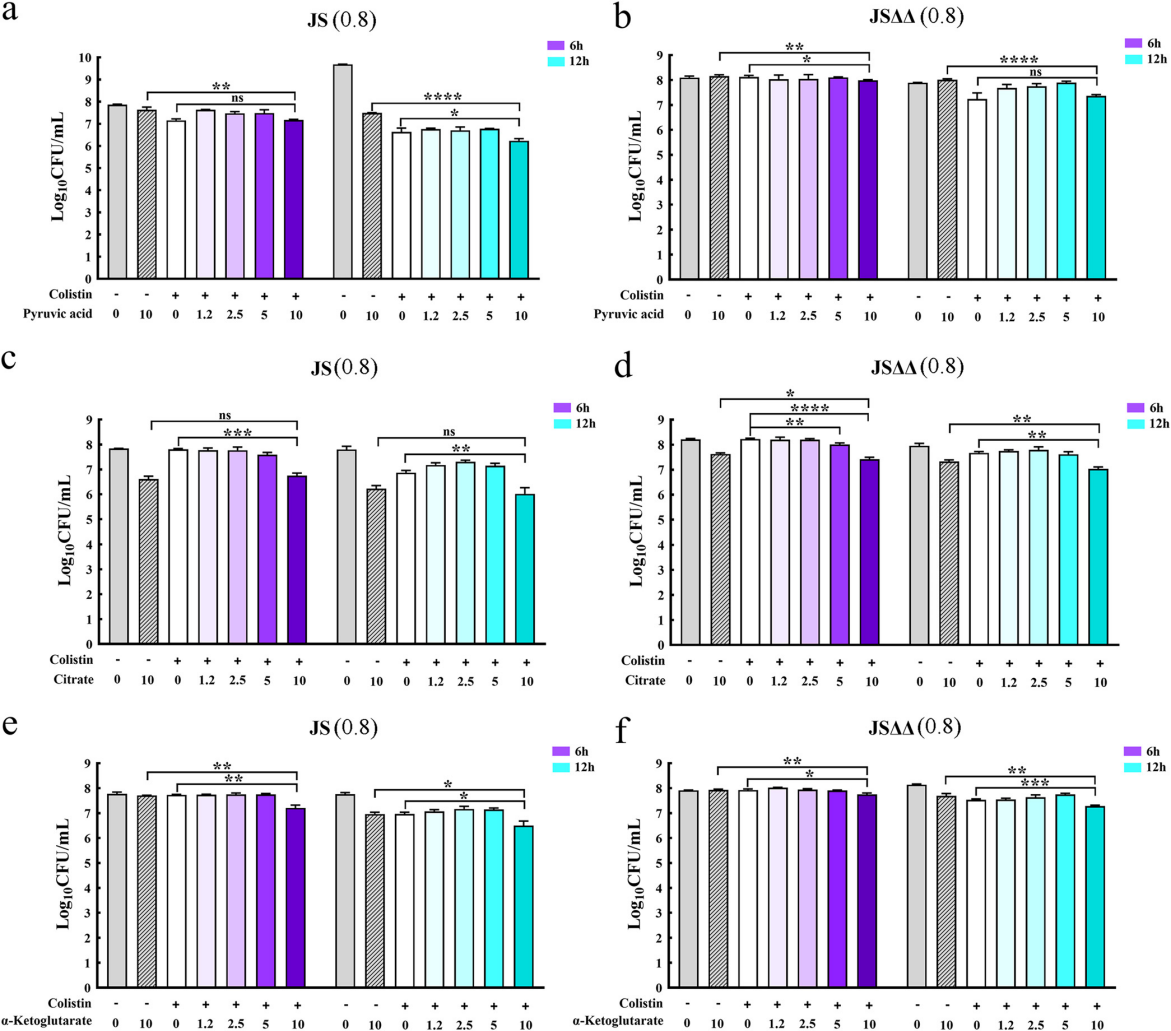
Bactericidal assay of JS, JSΔΔ in the presence of the indicated metabolites plus colistin. Cells were incubated with pyruvic acid (a and b), citrate (c and d) and α-ketoglutaric acid (e and f) in the presence or absence of colistin, respectively. Purple or blue color indicates that sampling time is 6 h or12 h, respectively, and color deepens with an increase in the concentration of metabolites. “+” and “-” represent that colistin (2 mg/L) or a metabolite (1.2, 2.5, 5, and 10 mM) were added or not. CFU were counted to estimate the number of viable bacteria. Statistically significant differences were indicated by asterisk * *P < *0.05, ** *P < *0.01, *** *P < *0.001, **** *P < *0.0001, and ns, no significant difference. Values in brackets represent the MICs of colistin for JS (0.8 mg/L) and JSΔΔ (0.8 mg/L).

Other studies have demonstrated that excess carbon sources such as fumarate, succinate, α-ketoglutarate, oxaloacetate, and pyruvate could restore susceptibility of pseudomonas aeruginosa, Edwardsiella tarda, or Vibrio alginolyticus to antibiotics (36–39). Mechanistic investigations showed that supplementation of these carbon sources sensitized cells to antibiotic killing by stimulating the TCA cycle, which may result in activation of respiratory metabolism, more generation of PMF. By contrast, Goossens et al. had reviewed that metabolic slowdown with reduced TCA cycle conferred a drug-tolerant phenotype in Mycobacterium tuberculosis (40). Thus, exogenous pyruvic acid, citrate, cis-aconitic acid, α-ketoglutaric acid, and (s)-malate were used in this study to test whether they could promote the colistin-mediated killing of JS and JSΔΔ strains. The survival of JS and JSΔΔ were both significantly reduced in the presence of colistin plus pyruvic acid, citrate, and α-ketoglutarate, and their antibacterial effects were enhanced successively by treatment of citrate, α-ketoglutarate, and pyruvic acid (Fig. 6), which were proportional to their fold changes in JSΔΔ/p*R*. While cis-aconitic acid and (s)-malate supplementation did not significantly alter the survival of JS (Fig. S3). The synergistic effects of these metabolites would be more significant, as the surviving cells may be re-growth after 6 h and 12 h of incubation. Therefore, we proposed that the significant accumulation of TCA cycle-associated metabolites, including citrate and α-ketoglutaric acid, in the more susceptible strain JSΔΔ/p*R* make contributions to the increased antibiotic lethality.

### Oxidative phosphorylation contributed little to enhance the colistin-mediated bacterial lethality.

**FIG 7:**
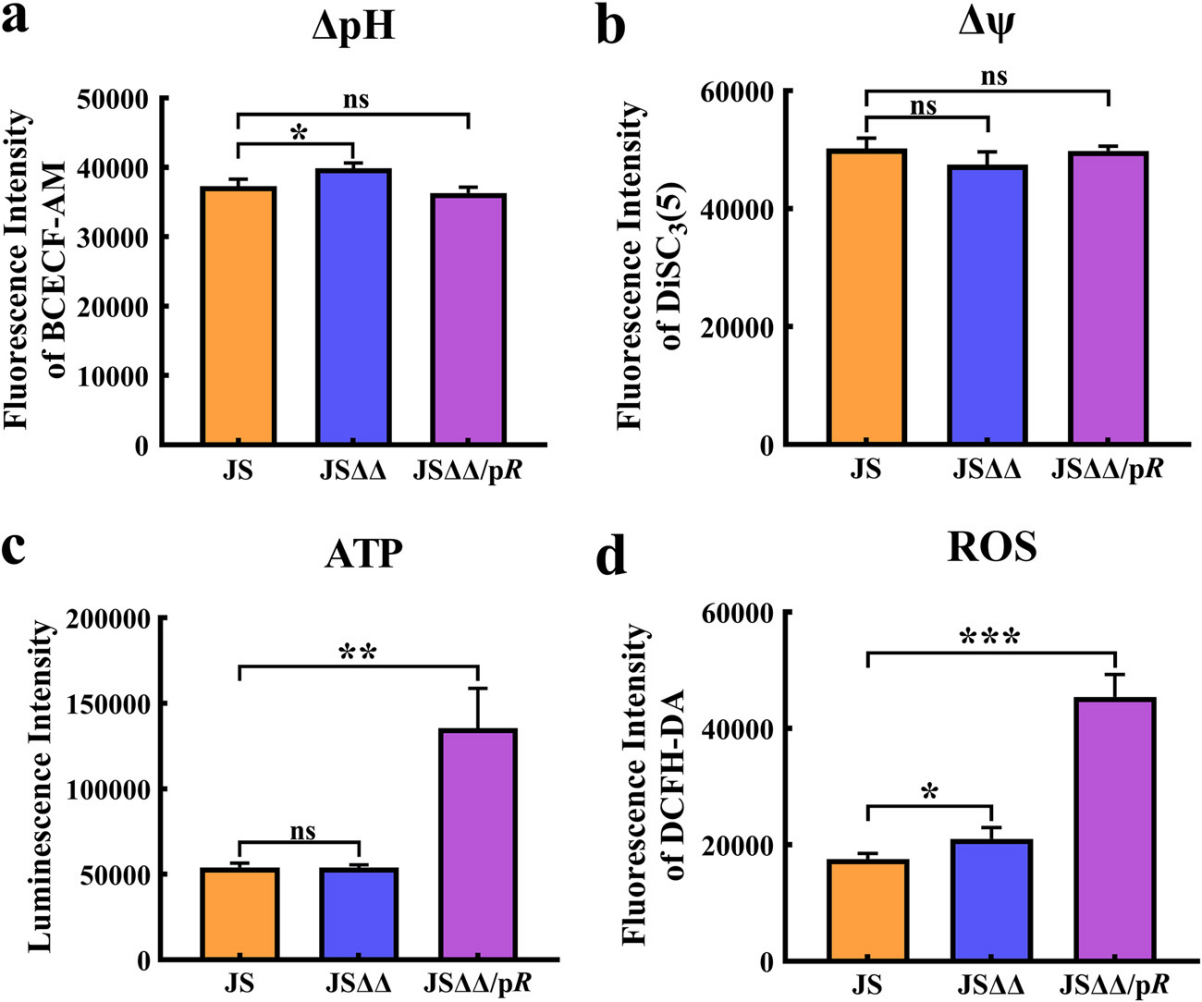
The PMF (a and b), intracellular ATP (c) and ROS (d) levels in of JS, JSΔΔ and JSΔΔ/p*R*. The PMF is reflected by two indexes ΔpH and Δψ. The levels of analyte were indicated by the corresponding fluorescence intensities. Statistically significant values are indicated with an asterisk * *P < *0.05, ** *P < *0.01, *** *P < *0.001, and ns, no significant difference. The MICs of colistin for JS, JSΔΔ, and JSΔΔ/p*R* were 0.8, 0.8, and 0.05 mg/L, respectively.

Oxidative phosphorylation can be a major source of ATP for the normal function of most cells. Three types of respiratory NADH dehydrogenases (NDH-1, NDH-2, and NQR) involving in oxidative phosphorylation have been identified in bacteria (41). NDH-1 is encoded by the *nuoABCDEFGHIJKLMN* operon, which using flavin mononucleotide (FMN) to transport electrons during NADH oxidation and PMF generation (42). As prior mentioned, the decline of PMF and ATP have been of great importance for the increased tolerance of antibiotic treatment (43, 44), and enhanced PMF is known to drive the uptake of aminoglycoside antibiotics (45). Unexpectedly, we detected opposite changes in the level of FMN (0.19-fold) and phosphoric acid (Pi, 0.24-fold) in JSΔΔ/p*R* compared to that of JSΔΔ (Fig. 5a). Additionally, we also found that the expression level of *nuoABCDEFGHIJKLMN* operon were downregulated by 1.2- to 2.8-fold in JSΔΔ/p*R* compared to that of JSΔΔ and JS. These results indicated that there was decreased oxidative phosphorylation and may also in consequence PMF and ATP deficiency in JSΔΔ/p*R*. We subsequently measured the PMF (ΔpH and Δψ) and intracellular ATP levels in JS, JSΔΔ and JSΔΔ/p*R*. Unexpectedly, we found no dramatic change of Δψ and ΔpH, but significant increase of ATP production in JSΔΔ/p*R* (Fig. 7a to c). These phenotypes may be explained by the fact that ATP generated by oxidative phosphorylation contributes little to the colistin susceptibility of Salmonella. As suggested earlier, ATP formed via oxidative phosphorylation is dispensable for growth of Salmonella, because the strains lacking *nuo*, *ndh*, or *atpB*-encoded subunits of ATP synthase recovered from nitrosative stress as efficiently as wild-type controls (46).

The NADH-driven ATP synthesis through the transfer of electrons always coupled with ROS production. ROS play an important role in the physiology and pathology of cells, and usually associated with antimicrobial-mediated lethality (47). We discovered a marked increase of ROS production in JSΔΔ/p*R* after examining the ROS levels in JS, JSΔΔ, JSΔΔ/p*R* (Fig. 7d). We suggest that AcrB deletion and CpxR overexpression may act synergistically to increase the ROS level. As it has been shown that activated AcrAB-TolC could suppress antibiotic-mediated intracellular ROS accumulation, and ROS levels were significantly increased when the Δ*acrB* strain was exposed to external stressful events (48, 49). Furthermore, there were reports that CpxR could bind directly or work with additional regulatory pathways, e.g., ArcA, to repress the transcription of *nuo* operon, and thereby trigger ROS based cell death (50, 51). Overall, we suggested that AcrB and CpxR could target the ATP and ROS generation, but not PMF production to potentiate antibiotic activity of colistin.

### Striking accumulations of agmatine sulfate and perturbation of nucleotide metabolism were considered to play roles in the increased colistin susceptibility as well decreased virulence of JSΔΔ/pR.

**FIG 8:**
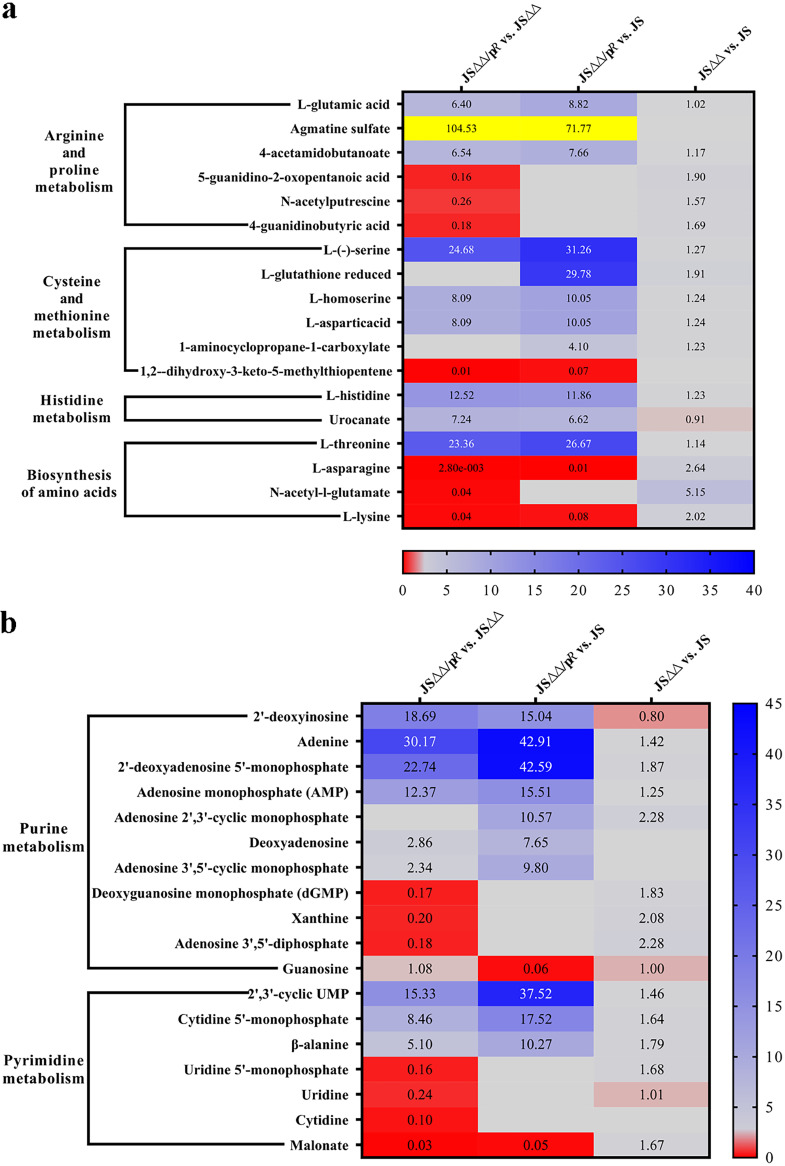
The SDMs detected in pathways of amino acids metabolism (a) and nucleotide metabolism (b) of JSΔΔ versus JS, JSΔΔ/p*R* versus JSΔΔ, and JSΔΔ/p*R* versus JS groups. Labels in each square indicate the fold changes of corresponding metabolites and squares without label indicate the data are not credible (*P > *0.05, VIP < 1). Background colors indicated the relative concentration of the respective metabolite or gene, red = decreased, dark/light blue = increased, yellow = strikingly increased. Gray background indicates indifferent concentrations with *P < *0.05, VIP ≥ 1. The MICs of colistin for JS, JSΔΔ, and JSΔΔ/p*R* were 0.8, 0.8, and 0.05 mg/L, respectively.

**FIG 9:**
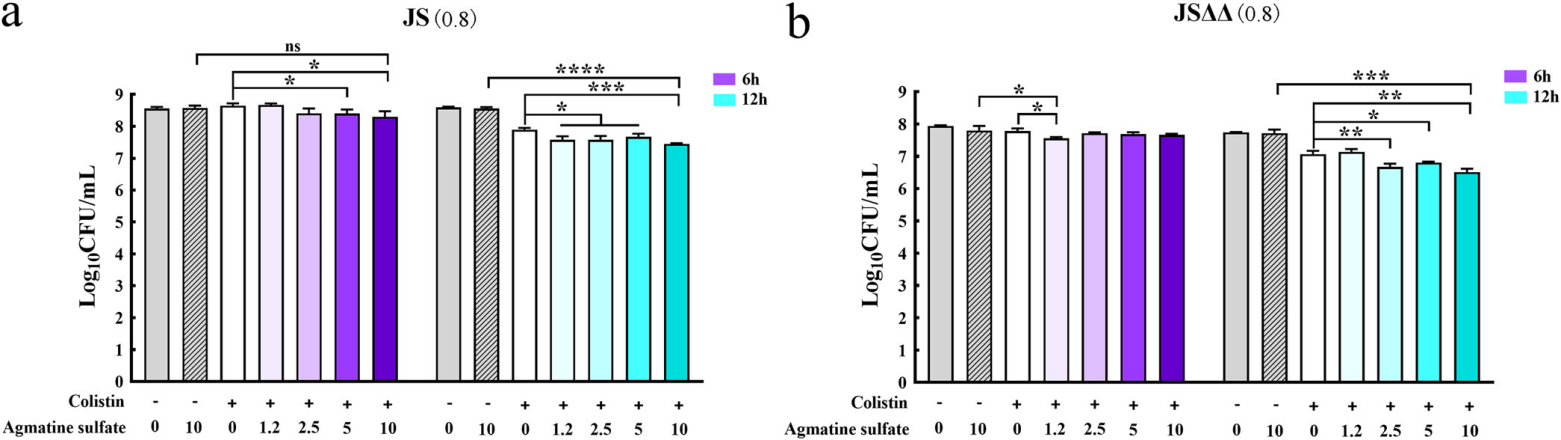
Bactericidal assay of JS, JSΔΔ in the presence of the agmatine sulfate plus colistin. Purple or blue color indicates that sampling time is 6 h or 12 h, respectively, and color deepens with an increase in the concentration of metabolites. “+” and “−” represent that colistin (2 mg/L) or agmatine sulfate (1.2, 2.5, 5, and 10 mM) were added or not. CFU were counted to estimate the number of viable bacteria. Statistically significant differences were indicated by asterisk * *P < *0.05, ** *P < *0.01, *** *P < *0.001, **** *P < *0.0001. Values in brackets represent the MICs of colistin for JS (0.8 mg/L) and JSΔΔ (0.8 mg/L).

Further alterations in the metabolic profile of JSΔΔ/p*R* were observed for amino acid concentrations (Fig. 8a). These include multiple pathways devoted to biosynthesis and metabolism of arginine, proline, cysteine, methionine, and histidine. Surprisingly, we observed an abnormally high agmatine sulfate content (104.53-fold) in JSΔΔ/p*R.* Meanwhile, exogenous agmatine sulfate could also drastically enhanced colistin bactericidal activity against JS and JSΔΔ in a concentration dependent manner (Fig. 9). Agmatine sulfate can be produced through the decarboxylation of arginine, and then converted to putrescine by agmatinase (52). Putrescine is ultimately metabolized by polyamine aminopropyltransferase, resulting in the production of polyamines, which have been reported previously to interact with anionic complexes such as DNA, RNA, ATP, and phospholipids, and then stimulate cell proliferation, gene expression for the survival of cells. Recent studies have also confirmed polyamines as potent antioxidants by blocking free radicals from binding DNA and inhibiting lipid peroxidation in cell membranes (53, 54). Thus, we considered that the accumulation of agmatine sulfate may be resulted from the downstream inhibition of polyamine production, which did not conducive for cell survival and enhanced the colistin-mediated killing of JSΔΔ/p*R*.

Nucleotide biosynthesis function in a variety of vital cellular and metabolic processes, such as synthesis of nucleic acids, energy production, delivering drugs (55). We have found in this study that both the purine and pyrimidine metabolism pathways were markedly changed in JSΔΔ/p*R* (Fig. 8b), and the intermediates involved in purine metabolism pathway (adenine, 2′-deoxyadenosine 5′-monophosphate, AMP) or pyrimidine metabolism pathway (2′,3′-cyclic UMP) were upregulated by approximately 15- to 40-fold. Yang et al. (56) have found that adenine supplementation in E. coli decreased ATP demand, which elevating metabolic activity of central carbon, and thus reduced the lethality of antibiotics. In contrast, pyrimidine supplementation, including uracil or cytosine, could inhibit pyrimidine biosynthesis, promote purine biosynthesis activity and increase the antibiotic lethality. However, the relationship between nucleotide biosynthesis and antibiotic was still controversial, since daptomycin-nonsusceptible Staphylococcus aureus showed increased levels of both purines and pyrimidines, while mutations in these pathways showed contrarily decreased formation of persister cells after treatment with rifampicin (57, 58). Nucleotide synthesis inhibitors (e.g., 6-thioguanine) have been developed for treatment of Staphylococcus aureus or Mycoplasma pneumoniae (59, 60). Of note, nucleotide biosynthesis pathways have strong links with full virulence of bacterial pathogens, and researchers have discovered that mutations in different pathway steps of nucleotide biosynthesis, including *carA*, *carB*, or *pyrD*, could consequently lead to decreased expression of virulence factors in Pseudomonas aeruginosa (61, 62). Hence, we suggested that the disruption of nucleotide metabolism after *acrB* deletion and *cpxR* overexpression played a role in the increased colistin susceptibility as well decreased virulence of JSΔΔ/p*R* in this paper.

### Conclusions.

Taken together, we have demonstrated that *acrB* deletion and *cpxR* overexpression have caused striking perturbations at both the transcriptomics and metabolomics levels in JSΔΔ/p*R*. The virulence-related genes and CRRGs were markedly downregulated in the more susceptible strain JSΔΔ/p*R*. The significant accumulation of metabolites in the TCA cycle and amino acid metabolism, including citrate, α-ketoglutaric acid, and agmatine sulfate, could potentiate the colistin-mediated killing of Salmonella, indicating that these metabolites may serve as potential adjuvants for colistin therapy. Additionally, we also suggested that AcrB and CpxR could synergistically target the ATP and ROS, but not PMF production to potentiate antibiotic activity of colistin. These results revealed several previously unknown regulatory mechanisms of AcrB and CpxR on the colistin susceptibility and provides a theoretical basis for finding potential new drug targets and adjuvants.

## MATERIALS AND METHODS

### Bacterial strains and growth conditions.

The *acrB* and *cpxR* double-deleted mutant JSΔ*acrB*Δ*cpxR*::*kan* (simplified as JSΔΔ) and corresponding *cpxR* complementary strain JSΔ*acrB*Δ*cpxR*::*kan*/p*cpxR* (simplified as JSΔΔ/p*R*) were generated from a multidrug-susceptible standard strain of Salmonella Typhimurium CVCC541 (named as JS) in our previous paper (63). The MICs of colistin for these strains were 0.8 mg/L (JS), 0.8 mg/L (JSΔΔ), and 0.05 mg/L (JSΔΔ/p*R*), respectively. For bacterial growth, single clones were propagated in LB medium and cultured to reach an OD_600_ of 0.6, then cells were washed 3 times and collected for further use.

### Transcriptome analysis.

Transcriptome sequencing and functional annotation in JS, JSΔΔ, and JSΔΔ/p*R* were carried out by Sangon Biotech (China). The clean reads were mapped to the Salmonella Typhimurium CT18 genome (NC003198) from NCBI using Bowtie2. We identified significantly differentially expressed genes (SDEGs) that with a qValue < 0.05 and |Fold Change| >2 (log_2_FC ≥ 1or ≤ −1). We further analyzed and compared the SDEGs between each pair of the three groups, i.e., JSΔΔ compared to JS (JSΔΔ versus JS), JSΔΔ/p*R* compared to JSΔΔ (JSΔΔ/p*R* versus JSΔΔ), or JSΔΔ/p*R* compared to JS (JSΔΔ/p*R* versus JS). The functional enrichment of SDEGs were annotated and classified by Gene Ontology (GO: http://geneontology.org) and Kyoto Encyclopedia of Genes and Genomes pathways (KEGG: http://www.kegg.jp). The relative transcription levels described in this paper referred to Log_2_FC of all transcripts.

### Lipid A characterization by ESI-MS.

Lipid A samples were prepared by the Bligh-Dyer method as previously described (64). For ESI-MS analysis, samples were dissolved with 1 mL solvent mixture of chloroform/methanol (4:1, vol/vol). The ESI-MS analysis were performed with the negative-ion mode of a Q Exactive Plus MALDI-TOF mass spectrometer (Thermo Fisher Scientific, USA).

### Metabolome analysis.

Metabolite extraction and untargeted metabolomics analysis were carried out by the Beijing Genomics Institute. PCA and partial least squares-discriminant analysis (PLS-DA) combined with univariate analysis (fold-change, FC) and Student's *t* test were used to screen the SDMs. SDMs (VIP ≥ 1, Fold-Change ≥ 1.2 or ≤0.83, *P < *0.05) were used for pathway analysis and identified by BGI Library (self-built library of Beijing Genomics Institute), mzCloud (http://www.chemspider.com), ChemSpider (http://www.chemspider.com), HMDB (http://www.hmdb.ca), KEGG (https://www.kegg.jp), and Lipidmaps (https://www.lipidmaps.org) databases.

### Colistin accumulation measured by LC-MS/MS.

Overnight cultures of JS, JSΔΔ, and JSΔΔ/p*R* were washed twice with 40 mL PBS, and pellets were resuspended in PBS and 900 μL each tube for three repetitions for each compound. Meanwhile, CFU were determined by the 10-fold serial broth microdilution method. Samples were equilibrated at 37°C with shaking for 5 min, and then 100 μL ciprofloxacin, novobiocin, and colistin were added (final concentrations were 1.5, 0.6, and 0.4 μmol/mL, respectively), with continually incubated for another 10 min. After incubation, 800 μL cultures were gingerly layered on 700 μL silicone oil (AR20/Sigma High Temperature), then centrifuged (9,400 rcf, 5 min) and removed the above supernatant and oil. After the resuspension in 200 μL water, each pellet were subjected to three freeze-thaw cycle of liquid nitrogen (3 min) in followed by 65°C water bath (3 min). Samples were pelleted at 9,400 rcf for 5 min and 180 μL supernatant was collected. Each pellet was resuspended in 100 μL methanol, and supernatant was combined with the previous supernatant collected after centrifuging (9,400 rcf, 5 min). Supernatants were 100 or 1,000 times diluted and analyzed by LC–MS/MS.

### Bactericidal assay *in vitro*.

Bacteria cells were prepared as described above and resuspended in LB media, adjusted OD_600_ to 0.5. Colistin (2 mg/L) or/and a metabolite (1.2, 2.5, 5, and 10 mM) were added and incubated at 37°C for 6 h or 12 h. After incubation, CFU were determined by the 10-fold serial broth microdilution method. The metabolites, including pyruvic acid, citrate, α-ketoglutaric acid, cis-aconitic acid, and (s)-malate and agmatine sulfate, were all purchased from Sigma-Aldrich and dissolved in water.

### PMF assay.

The pH-sensitive fluorescent probe BCECF-AM (20 μM) (Beyotime, Shanghai, China) and 3,3-dipropylthiadicarbocyanine iodide (DiSC_3_(5), 0.5 μM) (Aladdin, Shanghai, China) were applied respectively to determine the pH gradient (ΔpH) and membrane potential (Δψ). The fluorescence intensity was measured with the excitation wavelength at 500 nm, 622 nm and emission wavelength at 522 nm, 670 nm, respectively (65).

### Intracellular ATP and ROS levels assay.

Intracellular ATP and ROS were assessed with the Enhanced ATP assay kit (Beyotime, Shanghai, China) and Intracellular ROS assay kit (Beyotime, Shanghai, China), respectively. Bacterial precipitates were lysed using lysozyme, and centrifuged. Subsequently, the supernatant liquids were prepared for intracellular ATP and ROS measurement according to the manufacturer’s instructions.

### Statistical analysis.

Unless otherwise noted, all data were expressed as mean ± SEM (*n* = 3 biological replicates), and statistical analysis was performed using Student's *t* test. The * *P < *0.05, ** *P < *0.01, *** *P < *0.001, and **** *P < *0.0001 were considered statistically significant.

### Data availability.

Transcriptome data have been submitted to the Sequence Read Archive (SRA) database under the BioProject accession number PRJNA625887 (SRA accession numbers SAMN14610720, SAMN14610710, and SAMN14610595). The raw data for the metabolomics have been uploaded to the MetaboLights (www.ebi.ac.uk/metabolights) under accession number MTBLS7745. It is anticipated that this accession number will be released by 2 July 2023; until that time, the data will be available from the corresponding author upon request.
