# Supplementary material for: Analysis of Regulatory Mechanism of AcrB and CpxR on Colistin Susceptibility Based on Transcriptome and Metabolome of Salmonella Typhimurium
Source: Microbiol Spectr. 2023 Jun 26;11(4):e00530-23. doi: 10.1128/spectrum.00530-23 (PMC10434024; doi:10.1128/spectrum.00530-23)
Supplement: Supplemental file 1 — Supplemental material. Download spectrum.00530-23-s0001.docx, DOCX file, 1.2 MB [file spectrum.00530-23-s0001.docx]

**Supplementary materials for**

Title: Analysis of regulatory mechanism of AcrB and CpxR on colistin susceptibility based on transcriptome and metabolome of *Salmonella* Typhimurium

Ya-Jun Zhai,^a^ Pei-Yi Liu,^a^ Xing-Wei Luo,^a^ Jun Liang,^b^ Ya-Wei Sun,^c^ Xiao-Die Cui,^a^ Dan-Dan He,^a^ Yu-Shan Pan,^a^ Hua Wu^a^# and Gong-Zheng Hu^a^#

^a^College of Veterinary Medicine, Henan Agricultural University, 450002 Zhengzhou, China ^b^Zhengzhou Animal Husbandry Bureau, 450052 Zhengzhou, China

^c^Henan Institute of Science and Technology, 453003 Xinxiang, China

Running Head: AcrB and CpxR regulate the colistin susceptibility

#Address correspondence to Gong-Zheng Hu, [yaolilab@126.com](mailto:yaolilab@126.com), and Hua Wu, wuhua@henau.edu.cn

Ya-Jun Zhai, Pei-Yi Liu and Xing-Wei Luo contributed equally to this paper. The order of authors was arranged by the contribution they made to the current work.

**
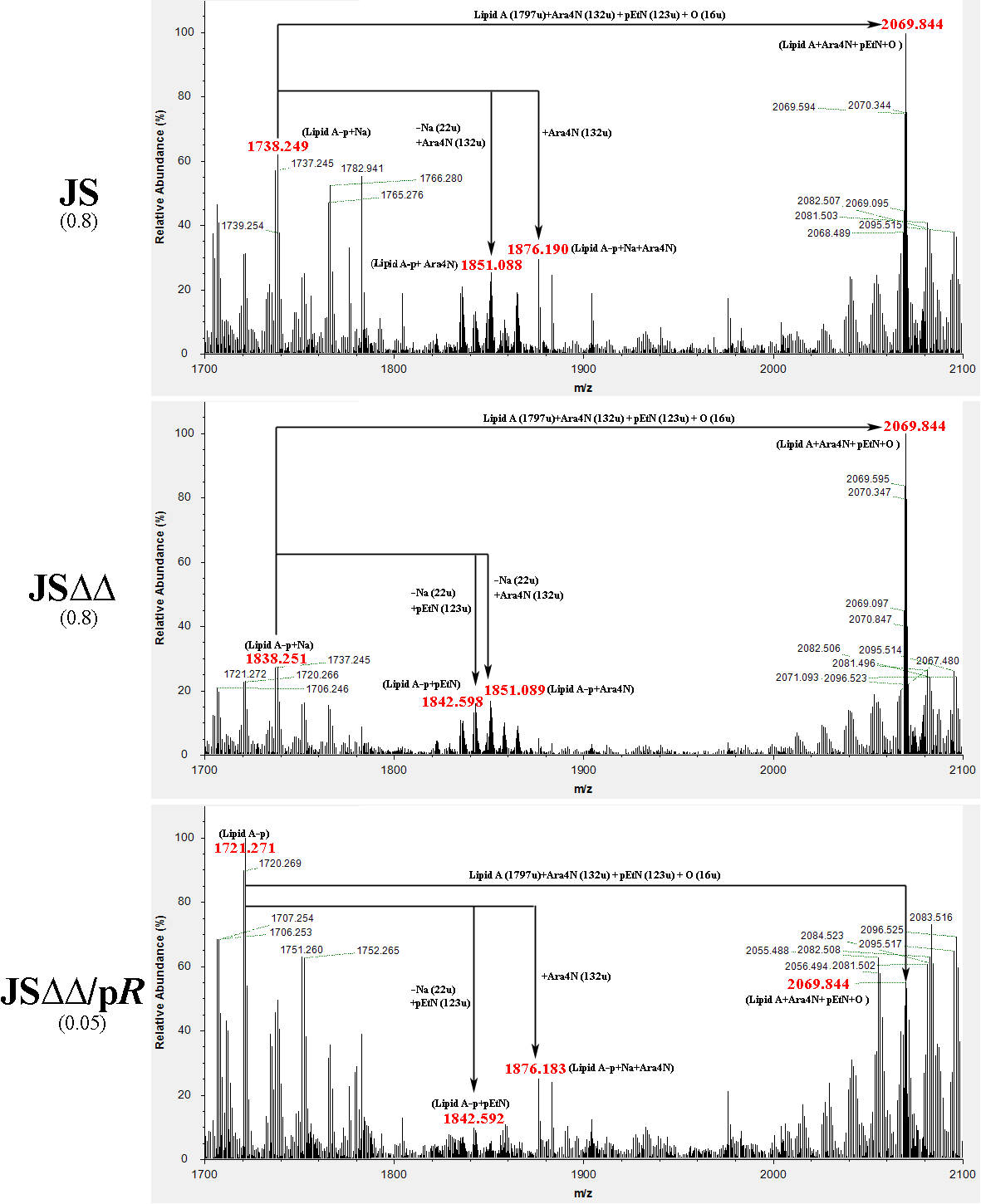
**

**Figure S1. ESI-MS analysis of lipid A species produced by JS, JS∆∆, and JS∆∆/p*R*.** The lipid A isolated from JS yielded peaks at m/z 1738.249, 1851.088, 1876.190 and 2069.844. The peak at *m/z* 1738.249 were interpreted as a single-phosphorylated hexa-acylated structure with Na^+^ (*m/z* = 1738.249; i.e., 1797.2 - 80 + 22). Peaks at *m/z* 1851.088 and 1876.190 were likely represented single-phosphorylated hexa-acylated lipid A with the addition of L-Ara4N with or without Na^+^ (*m/z* = 1851.088; i.e., 1738.249 + 132; *m/z* = 1876.190; i.e., 1738.249 + 132 + 22). The peak at *m/z* 2069.844 indicated the addition of L-Ara4N, pEtN and a hydroxylation to the bis-phosphorylated exah-acylated lipid A (*m/z =* 2069.844; i.e., 1738.249 + 132 + 123 + 16). The lipid A isolated from JS∆∆ showed a relatively lower abundance of 1738.249, and one additional peak at *m/z* 1842.598 (pEtN single-modified lipid A; *m/z* = 1842.598; i.e.,1738.249 + 123) compared with JS. Mass spectra of colistin-susceptible strain JS∆∆/p*R* was characterized by one additional high peak at m/z 1721.271 compared with JS and JS∆∆, which represent the bis-phosphorylated hexa-acylated lipid A and the removal of phosphate (*m/z =* 1721.2271; i.e., 1797.2 - 80). Furthormore, the lipid A in JS∆∆/p*R* showed lower abundance of pEtN and L-Ara4N modification at *m/z* 1842.598 and 2069.844 compared with that of JS and JS∆∆. Values in brackets represent the MICs of colistin for JS (0.8 mg/L), JS∆∆ (0.8 mg/L) and JS∆∆/p*R* (0.05 mg/L).

**
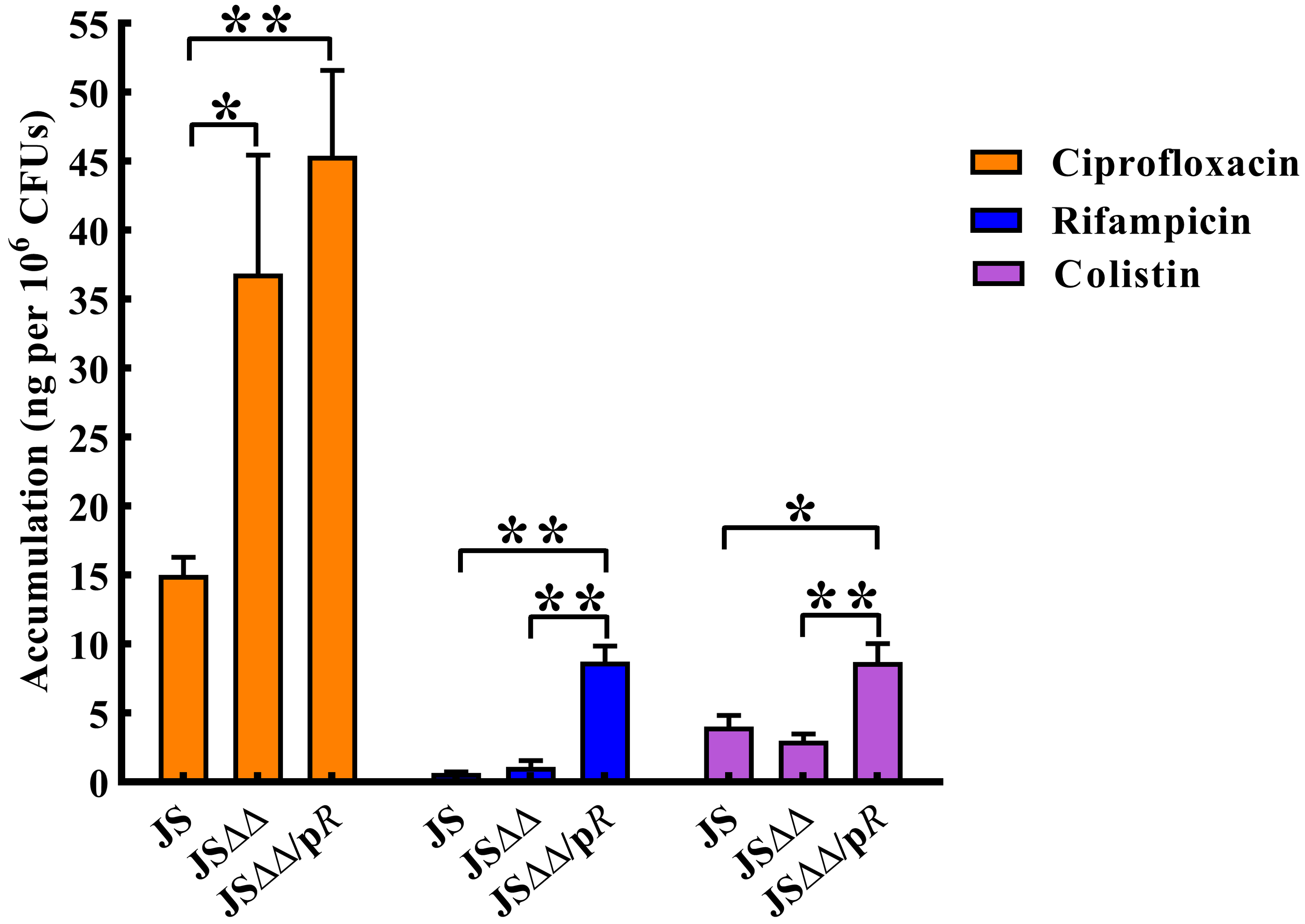
**

**Figure S2. Colistin accumulation in JS, JS∆∆, and JS∆∆/p*R*.** As control groups, the high/low-accumulating antibiotics ciprofloxacin/[rifampicin](javascript:;) were also measured. Statistical significance was determined by using Student’s t-tests and asterisks indicate a significant difference (* *p* < 0.05, ** *p* < 0.01) compared with the accumulating antibiotics of JS or JS∆∆. The MICs of colistin for JS, JS∆∆ and JS∆∆/p*R* were 0.8, 0.8 and 0.05 mg/L, respectively.

**
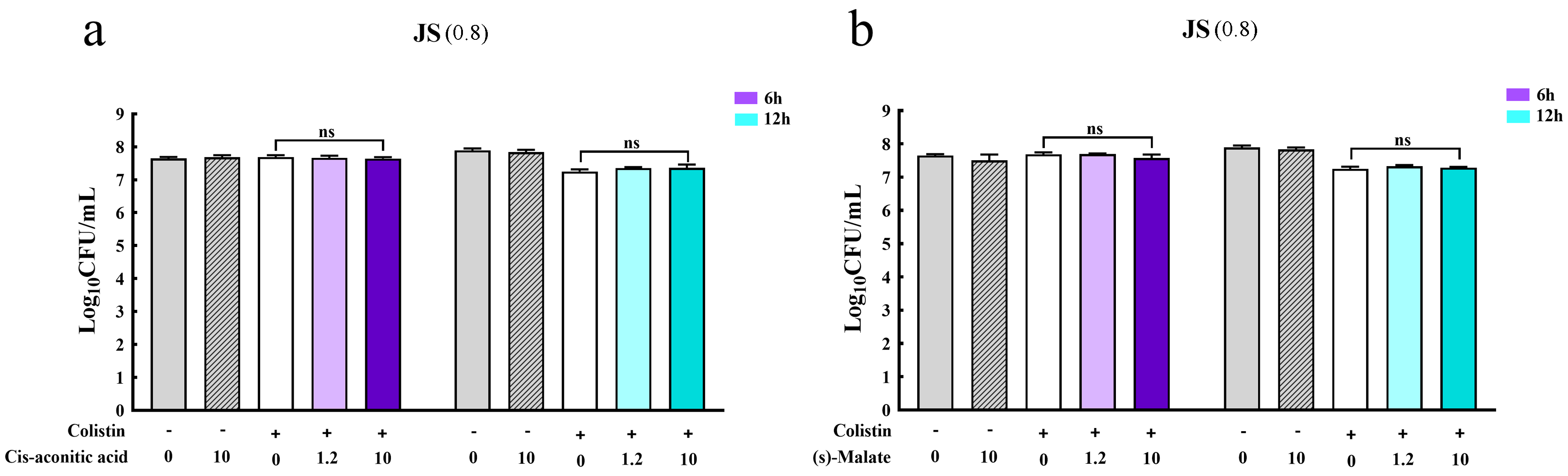
**

**Figure S3. Bactericidal assay of JS in the presence of the cis-aconitic acid (a) or (s)-malate (b) plus colistin.** Purple or blue color indicates that sampling time is 6h or12h, respectively, and color deepens with an increase in the concentration of metabolites. “+” and “-” represent that colistin (2 mg/L) or a metabolite (1.2 and 10 mM) were added or not. CFUs were counted to estimate the number of viable bacteria. The “ns” represents no significant difference. Values in brackets represent the MICs of colistin for JS (0.8 mg/L).

**Table S1 The top 20 up-regulated and down-regulated genes in JS∆∆ vs. JS, JS∆∆/p*R* vs. JS∆∆ and JS∆∆/p*R* vs. JS groups**

| **Groups** | **Gene ID** | **Name** | **Log_2_FC** | **Result** | **Description** |
| --- | --- | --- | --- | --- | --- |
| JS∆∆ Vs. JS | STY2243 | *pduA* | 22.47 | up | propanediol utilization protein PduA |
| JS∆∆ Vs. JS | STY0669 | STY0669 | 16.76 | up | apo-citrate lyase phosphoribosyl-dephospho-CoA transferase |
| JS∆∆ Vs. JS | STY2246 | *pduD* | 10.33 | up | diol dehydratase medium subunit |
| JS∆∆ Vs. JS | STY2244 | *pduB* | 9.90 | up | propanediol utilization protein PduB |
| JS∆∆ Vs. JS | STY2245 | *pduC* | 9.32 | up | glycerol dehydratase large subunit |
| JS∆∆ Vs. JS | STY2247 | *pduE* | 8.77 | up | diol dehydratase small subunit |
| JS∆∆ Vs. JS | STY2252 | *pduL* | 7.69 | up | propanediol utilization phosphotransacylase |
| JS∆∆ Vs. JS | STY2248 | *pduG* | 7.55 | up | propanediol utilization diol dehydratase reactivation protein PduG |
| JS∆∆ Vs. JS | STY2249 | *pduH* | 6.87 | up | propanediol dehydratase reactivation protein PduH |
| JS∆∆ Vs. JS | STY2251 | *pduK* | 6.78 | up | propanediol utilization protein PduK |
| JS∆∆ Vs. JS | STY2255 | *pduO* | 6.76 | up | propanediol utilization protein PduO |
| JS∆∆ Vs. JS | STY2257 | *pduQ* | 6.42 | up | propanol dehydrogenase |
| JS∆∆ Vs. JS | STY2256 | *pduP* | 6.33 | up | CoA-dependent proprionaldehyde dehydrogenase |
| JS∆∆ Vs. JS | STY2253 | *pduM* | 6.21 | up | propanediol utilization protein PduM |
| JS∆∆ Vs. JS | STY2250 | *pduJ* | 6.06 | up | propanediol utilization protein PduJ |
| JS∆∆ Vs. JS | STY2259 | *pduT* | 5.59 | up | propanediol utilization protein PduT |
| JS∆∆ Vs. JS | STY2258 | *pduS* | 4.92 | up | ferredoxin |
| JS∆∆ Vs. JS | STY0672 | *citD* | 4.60 | up | citrate lyase acyl carrier protein |
| JS∆∆ Vs. JS | STY3664 | STY3664 | 4.30 | up | hypothetical protein |
| JS∆∆ Vs. JS | STY4025 | STY4025 | 4.25 | up | hydrolase |
| JS∆∆ Vs. JS | STYt018 | STYt018 | -17.13 | down | tRNA-Val |
| JS∆∆ Vs. JS | STY3811 | *cpxP* | -10.60 | down | cell-envelope stress modulator CpxP |
| JS∆∆ Vs. JS | STY2703 | STY2703 | -7.04 | down | cobalamin adenosyltransferase |
| JS∆∆ Vs. JS | STY2706 | *eutS* | -7.00 | down | ethanolamine utilization protein EutS |
| JS∆∆ Vs. JS | STY2705 | STY2705 | -6.98 | down | ethanolamine utilization protein EutP |
| JS∆∆ Vs. JS | STY2698 | *eutG* | -6.79 | down | alchohol dehydrogenase |
| JS∆∆ Vs. JS | STY2702 | *eutD* | -6.79 | down | phosphate acyltransferase |
| JS∆∆ Vs. JS | STY2704 | STY2704 | -6.67 | down | ethanolamine utilization protein EutQ |
| JS∆∆ Vs. JS | STY2701 | *eutN* | -6.40 | down | ethanolamine utilization protein EutN |
| JS∆∆ Vs. JS | STY3812 | *cpxR* | -6.28 | down | two-component response regulatory protein CpxR |
| JS∆∆ Vs. JS | STY2700 | STY2700 | -6.16 | down | aldehyde dehydrogenase |
| JS∆∆ Vs. JS | STY2699 | STY2699 | -6.07 | down | ethanolamine utilization protein EutJ |
| JS∆∆ Vs. JS | STY2696 | *eutA* | -5.43 | down | ethanolamine utilization protein EutA |
| JS∆∆ Vs. JS | STY2694 | *eutC* | -5.08 | down | ethanolamine ammonia-lyase light chain |
| JS∆∆ Vs. JS | STY2695 | *eutB* | -5.06 | down | ethanolamine ammonia-lyase heavy chain |
| JS∆∆ Vs. JS | STY2697 | STY2697 | -4.96 | down | ethanolamine utilization protein EutH |
| JS∆∆ Vs. JS | STY3726a | STY3726a | -4.16 | down | hypothetical protein |
| JS∆∆ Vs. JS | STY2692 | *eutK* | -3.99 | down | ethanolamine utilization protein EutK |
| JS∆∆ Vs. JS | STY2691 | STY2691 | -3.92 | down | ethanolamine operon transcriptional regulator |
| JS∆∆ Vs. JS | STY2693 | *eutL* | -3.75 | down | ethanolamine utilization protein EutL |
|  |  |  |  |  |  |
| JS∆∆/p*R* Vs. JS | STY0373 | *stbA* | 17.85 | up | fimbrial protein |
| JS∆∆/p*R* Vs. JS | STY2930 | *nrdH* | 16.27 | up | glutaredoxin |
| JS∆∆/p*R* Vs. JS | HCM1.216 | *bla* | 10.18 | up | beta-lactamase |
| JS∆∆/p*R* Vs. JS | STY1805 | STY1805 | 7.61 | up | hypothetical protein |
| JS∆∆/p*R* Vs. JS | STY3179 | *lomR* | 7.32 | up | Virulence-related outer membrane protein |
| JS∆∆/p*R* Vs. JS | STY0860 | STY0860 | 7.11 | up | hypothetical protein |
| JS∆∆/p*R* Vs. JS | STY2931 | *nrdI* | 6.92 | up | ribonucleotide reductase stimulatory protein |
| JS∆∆/p*R* Vs. JS | STY3557 | STY3557 | 6.66 | up | sulfite oxidase subunit YedY |
| JS∆∆/p*R* Vs. JS | STY3178 | STY3178 | 6.21 | up | hypothetical protein |
| JS∆∆/p*R* Vs. JS | STY3558 | STY3558 | 6.15 | up | sulfite oxidase subunit YedZ |
| JS∆∆/p*R* Vs. JS | STY0747 | STY0747 | 6.02 | up | potassium-transporting ATPase subunit A |
| JS∆∆/p*R* Vs. JS | STY3542 | STY3542 | 5.81 | up | hypothetical protein |
| JS∆∆/p*R* Vs. JS | STY3074 | STY3074 | 5.72 | up | 3'-phosphoadenosine 5'-phosphosulfate sulfotransferase |
| JS∆∆/p*R* Vs. JS | STY2933 | STY2933 | 5.53 | up | ribonucleoside-diphosphate reductase 2 subunit beta |
| JS∆∆/p*R* Vs. JS | STY1540 | STY1540 | 5.46 | up | multiple antibiotic resistance protein MarR |
| JS∆∆/p*R* Vs. JS | STY3076 | STY3076 | 5.43 | up | sulfite reductase (NADPH) flavoprotein subunit beta |
| JS∆∆/p*R* Vs. JS | STY0624 | *ramA* | 5.40 | up | transcriptional activator RamA |
| JS∆∆/p*R* Vs. JS | STY2437 | STY2437 | 5.35 | up | hypothetical protein |
| JS∆∆/p*R* Vs. JS | STY2805 | STY2805 | 5.24 | up | cadaverine/lysine antiporter |
| JS∆∆/p*R* Vs. JS | STY3075 | STY3075 | 5.21 | up | sulfite reductase (NADPH) hemoprotein subunit alpha |
| JS∆∆/p*R* Vs. JS | STYt021 | STYt021 | -18.92 | down | tRNA-Arg |
| JS∆∆/p*R* Vs. JS | STY3016 | *spaM* | -18.67 | down | secretory protein |
| JS∆∆/p*R* Vs. JS | STY3328 | STY3328 | -17.59 | down | hypothetical protein |
| JS∆∆/p*R* Vs. JS | STY0600 | *fimW* | -16.89 | down | fimbriae w protein |
| JS∆∆/p*R* Vs. JS | STY0196 | STY0196 | -16.15 | down | PTS system transporter subunit IIA |
| JS∆∆/p*R* Vs. JS | STY3870 | STY3870 | -15.62 | down | hydrolase |
| JS∆∆/p*R* Vs. JS | STY3022 | *invF* | -12.43 | down | AraC family transcriptional regulator |
| JS∆∆/p*R* Vs. JS | STY2704 | STY2704 | -11.48 | down | ethanolamine utilization protein EutQ |
| JS∆∆/p*R* Vs. JS | STY2705 | STY2705 | -10.90 | down | ethanolamine utilization protein EutP |
| JS∆∆/p*R* Vs. JS | STY2706 | *eutS* | -10.74 | down | ethanolamine utilization protein EutS |
| JS∆∆/p*R* Vs. JS | STY3020 | *invE* | -10.62 | down | cell invasion protein |
| JS∆∆/p*R* Vs. JS | STY2703 | STY2703 | -10.29 | down | cobalamin adenosyltransferase |
| JS∆∆/p*R* Vs. JS | STY3021 | *invG* | -10.24 | down | secretory protein |
| JS∆∆/p*R* Vs. JS | STY3006 | *sipD* | -10.22 | down | pathogenicity island 1 effector protein |
| JS∆∆/p*R* Vs. JS | STY3005 | *sipA* | -10.09 | down | pathogenicity island 1 effector protein |
| JS∆∆/p*R* Vs. JS | STY3007 | *sipC* | -10.03 | down | pathogenicity island 1 effector protein |
| JS∆∆/p*R* Vs. JS | STY2993 | *prgJ* | -9.95 | down | pathogenicity 1 island effector protein |
| JS∆∆/p*R* Vs. JS | STY0589 | *fimA* | -9.95 | down | type-1 fimbrial protein subunit A |
| JS∆∆/p*R* Vs. JS | STY3008 | *sipB* | -9.83 | down | pathogenicity island 1 effector protein |
| JS∆∆/p*R* Vs. JS | STY3009 | STY3009 | -9.74 | down | chaperone protein SicA |
|  |  |  |  |  |  |
| JS∆∆/p*R* vs. JS∆∆ | STY2930 | *nrdH* | 16.27 | up | glutaredoxin |
| JS∆∆/p*R* vs. JS∆∆ | STY3811 | *cpxP* | 11.94 | up | cell-envelope stress modulator CpxP |
| JS∆∆/p*R* vs. JS∆∆ | HCM1.216 | *bla* | 10.42 | up | beta-lactamase |
| JS∆∆/p*R* vs. JS∆∆ | STY3179 | *lomR* | 8.08 | up | Virulence-related outer membrane protein |
| JS∆∆/p*R* vs. JS∆∆ | STY0860 | STY0860 | 7.13 | up | hypothetical protein |
| JS∆∆/p*R* vs. JS∆∆ | STY3726a | STY3726a | 6.82 | up | hypothetical protein |
| JS∆∆/p*R* vs. JS∆∆ | STY1975 | *htpX* | 6.69 | up | protease HtpX |
| JS∆∆/p*R* vs. JS∆∆ | STY1805 | STY1805 | 6.59 | up | hypothetical protein |
| JS∆∆/p*R* vs. JS∆∆ | STY3557 | STY3557 | 6.43 | up | sulfite oxidase subunit YedY |
| JS∆∆/p*R* vs. JS∆∆ | STY3178 | STY3178 | 6.41 | up | hypothetical protein |
| JS∆∆/p*R* vs. JS∆∆ | STY1149 | *scsA* | 6.16 | up | copper-sensitivity supressor protein A |
| JS∆∆/p*R* vs. JS∆∆ | STY1461 | STY1461 | 6.02 | up | hypothetical protein |
| JS∆∆/p*R* vs. JS∆∆ | STY3558 | STY3558 | 5.88 | up | sulfite oxidase subunit YedZ |
| JS∆∆/p*R* vs. JS∆∆ | STY2437 | STY2437 | 5.83 | up | hypothetical protein |
| JS∆∆/p*R* vs. JS∆∆ | STY3812 | *cpxR* | 5.60 | up | two-component response regulatory protein CpxR |
| JS∆∆/p*R* vs. JS∆∆ | STY4495 | *adi* | 5.41 | up | arginine decarboxylase |
| JS∆∆/p*R* vs. JS∆∆ | STY0747 | STY0747 | 5.37 | up | potassium-transporting ATPase subunit A |
| JS∆∆/p*R* vs. JS∆∆ | STY2931 | *nrdI* | 5.15 | up | ribonucleotide reductase stimulatory protein |
| JS∆∆/p*R* vs. JS∆∆ | STY0102 | STY0102 | 5.08 | up | dihydrofolate reductase type I |
| JS∆∆/p*R* vs. JS∆∆ | STY2086 | STY2086 | 4.90 | up | hypothetical protein |
| JS∆∆/p*R* vs. JS∆∆ | STYt021 | STYt021 | -18.83 | down | tRNA-Arg |
| JS∆∆/p*R* vs. JS∆∆ | STY0600 | *fimW* | -17.67 | down | fimbriae w protein |
| JS∆∆/p*R* vs. JS∆∆ | STY3016 | *spaM* | -17.64 | down | secretory protein |
| JS∆∆/p*R* vs. JS∆∆ | STY3328 | STY3328 | -17.43 | down | hypothetical protein |
| JS∆∆/p*R* vs. JS∆∆ | STYt031 | STYt031 | -16.58 | down | tRNA-Arg |
| JS∆∆/p*R* vs. JS∆∆ | STY0196 | STY0196 | -15.92 | down | PTS system transporter subunit IIA |
| JS∆∆/p*R* vs. JS∆∆ | STY0589 | *fimA* | -12.02 | down | type-1 fimbrial protein subunit A |
| JS∆∆/p*R* vs. JS∆∆ | STY3022 | *invF* | -11.28 | down | AraC family transcriptional regulator |
| JS∆∆/p*R* vs. JS∆∆ | STY2243 | *pduA* | -9.57 | down | propanediol utilization protein PduA |
| JS∆∆/p*R* vs. JS∆∆ | STY3020 | *invE* | -9.55 | down | cell invasion protein |
| JS∆∆/p*R* vs. JS∆∆ | STY3021 | *invG* | -9.32 | down | secretory protein |
| JS∆∆/p*R* vs. JS∆∆ | STY2993 | *prgJ* | -8.96 | down | pathogenicity 1 island effector protein |
| JS∆∆/p*R* vs. JS∆∆ | STY4870 | STY4870 | -8.74 | down | hypothetical protein |
| JS∆∆/p*R* vs. JS∆∆ | STY0592 | *fimC* | -8.66 | down | fimbrial chaperone protein |
| JS∆∆/p*R* vs. JS∆∆ | STY2994 | *prgI* | -8.62 | down | pathogenicity 1 island effector protein |
| JS∆∆/p*R* vs. JS∆∆ | STY3019 | *invA* | -8.61 | down | secretory protein |
| JS∆∆/p*R* vs. JS∆∆ | STY3005 | *sipA* | -8.61 | down | pathogenicity island 1 effector protein |
| JS∆∆/p*R* vs. JS∆∆ | STY4805 | STY4805 | -8.61 | down | arginine deiminase |
| JS∆∆/p*R* vs. JS∆∆ | STY1852 | *aroQ* | -8.57 | down | chorismate mutase |
| JS∆∆/p*R* vs. JS∆∆ | STY3006 | *sipD* | -8.51 | down | pathogenicity island 1 effector protein |

Note: The red font indicated SDMs involved in lipid A modification, virulence, and metabolism of bacteria.

**Table S2 List of metabolites in measured groups**

| **Mode** | **Group** | **Total** | **Up-regulated** | **Down--regulated** | **SDMs** |
| --- | --- | --- | --- | --- | --- |
| pos | JS∆∆ vs. JS | 580 | 214 | 366 | 180 |
|  | JS∆∆/p*R* vs. JS | 1814 | 1039 | 775 | 695 |
|  | JS∆∆/p*R* vs. JS∆∆ | 1943 | 1193 | 750 | 733 |
|  |  |  |  |  |  |
| neg | JS∆∆ vs. JS | 53 | 17 | 36 | 19 |
|  | JS∆∆/p*R* vs. JS | 351 | 206 | 145 | 148 |
|  | JS∆∆/p*R* vs. JS∆∆ | 359 | 247 | 112 | 148 |

**Table S3 List of the 45 metabolic pathways in JS∆∆/p*R* compared with JS or JS∆∆**

| **Pathway name** | **Pathway ID** | **JS∆∆/p*R* vs. JS∆∆** | | **JS∆∆/p*R* vs. JS** | | **JS∆∆ vs. JS** | | **Total** |
| --- | --- | --- | --- | --- | --- | --- | --- | --- |
|  |  | **neg** | **pos** | **neg** | **pos** | **neg** | **pos** |  |
| Tricarboxylic acid (TCA) cycle | map00020 | 3 | 3 | 2 | 3 | 0 | 0 | 20 |
| Oxidative phosphorylation | map00190 | 0 | 2 | 0 | 0 | 0 | 0 | 16 |
| Arginine and proline metabolism | map00330 | 0 | 7 | 0 | 4 | 0 | 0 | 78 |
| Cysteine and methionine metabolism | map00270 | 2 | 6 | 2 | 7 | 0 | 0 | 61 |
| Biosynthesis of amino acids | map01230 | 4 | 11 | 4 | 10 | 0 | 0 | 128 |
| Histidine metabolism | map00340 | 0 | 5 | 2 | 5 | 1 | 0 | 47 |
| Purine metabolism | map00230 | 0 | 7 | 3 | 7 | 0 | 2 | 95 |
| Pyrimidine metabolism | map00240 | 2 | 5 | 3 | 3 | 0 | 0 | 65 |
| Carbon metabolism | map01200 | 8 | 6 | 6 | 6 | 0 | 0 | 112 |
| Pentose phosphate pathway | map00030 | 5 | 0 | 3 | 0 | 0 | 0 | 35 |
| Glycine, serine and threonine metabolism | map00260 | 5 | 5 | 5 | 5 | 0 | 0 | 50 |
| Glyoxylate and dicarboxylate metabolism | map00630 | 5 | 5 | 4 | 5 | 0 | 0 | 62 |
| Methane metabolism | map00680 | 5 | 0 | 5 | 0 | 0 | 0 | 84 |
| ABC transporters | map02010 | 5 | 7 | 4 | 7 | 0 | 0 | 126 |
| Monobactam biosynthesis | map00261 | 3 | 3 | 3 | 3 | 0 | 0 | 39 |
| Aminoacyl-tRNA biosynthesis | map00970 | 3 | 6 | 3 | 6 | 0 | 0 | 52 |
| Valine, leucine and isoleucine biosynthesis | map00290 | 2 | 0 | 2 | 0 | 0 | 0 | 23 |
| Pyruvate metabolism | map00620 | 2 | 0 | 2 | 0 | 0 | 0 | 31 |
| beta-Alanine metabolism | map00410 | 2 | 4 | 3 | 4 | 0 | 0 | 32 |
| C5-Branched dibasic acid metabolism | map00660 | 2 | 4 | 0 | 4 | 0 | 0 | 34 |
| Butanoate metabolism | map00650 | 2 | 4 | 0 | 4 | 0 | 0 | 42 |
| Fatty acid biosynthesis | map00061 | 2 | 0 | 2 | 0 | 0 | 0 | 50 |
| Phosphotransferase system (PTS) | map02060 | 2 | 4 | 3 | 5 | 0 | 0 | 56 |
| Phenylalanine metabolism | map00360 | 2 | 3 | 2 | 3 | 0 | 0 | 72 |
| Bacterial chemotaxis | map02030 | 1 | 0 | 0 | 1 | 0 | 0 | 6 |
| D-Alanine metabolism | map00473 | 1 | 0 | 1 | 1 | 0 | 0 | 6 |
| One carbon pool by folate | map00670 | 1 | 0 | 1 | 0 | 0 | 0 | 9 |
| 2-Oxocarboxylic acid metabolism | map01210 | 2 | 7 | 0 | 6 | 0 | 0 | 134 |
| Taurine and hypotaurine metabolism | map00430 | 1 | 3 | 1 | 3 | 0 | 0 | 22 |
| Sphingolipid metabolism | map00600 | 1 | 0 | 1 | 0 | 0 | 1 | 25 |
| Alanine, aspartate and glutamate metabolism | map00250 | 0 | 6 | 0 | 6 | 0 | 0 | 28 |
| Tryptophan metabolism | map00380 | 0 | 8 | 0 | 6 | 0 | 0 | 81 |
| Arginine biosynthesis | map00220 | 0 | 4 | 0 | 3 | 0 | 1 | 23 |
| Pantothenate and CoA biosynthesis | map00770 | 0 | 4 | 2 | 3 | 0 | 1 | 28 |
| Sulfur metabolism | map00920 | 0 | 4 | 0 | 2 | 0 | 1 | 33 |
| Lysine biosynthesis | map00300 | 0 | 4 | 0 | 4 | 0 | 0 | 35 |
| Cyanoamino acid metabolism | map00460 | 0 | 4 | 0 | 4 | 0 | 0 | 45 |
| Two-component system | map02020 | 0 | 4 | 0 | 3 | 0 | 0 | 53 |
| Vitamin B6 metabolism | map00750 | 0 | 3 | 0 | 4 | 0 | 0 | 28 |
| D-Glutamine and D-glutamate metabolism | map00471 | 0 | 2 | 0 | 2 | 0 | 0 | 12 |
| Lysine degradation | map00310 | 0 | 3 | 0 | 4 | 0 | 0 | 55 |
| Biotin metabolism | map00780 | 0 | 2 | 0 | 2 | 0 | 0 | 28 |
| Glycolysis/Gluconeogenesis | map00010 | 0 | 2 | 0 | 2 | 0 | 0 | 31 |
| Thiamine metabolism | map00730 | 0 | 2 | 0 | 2 | 0 | 0 | 31 |
| Tyrosine metabolism | map00350 | 0 | 3 | 0 | 4 | 0 | 0 | 78 |

Note: The red font indicated the 8 pathways that changed both at transcriptional and metabolic levels.
